# Active surveillance of immunization adverse effects: a multicentre, open-label, three-arm randomized uncontrolled trial in Ethiopia

**DOI:** 10.1093/inthealth/ihae040

**Published:** 2024-06-03

**Authors:** Dawit Getachew Assefa, Tizalegn Tesefaye, Etaferaw Bekele, Genet Geberemickeal, Andualem Mebratu, Aschalew Gossaye Ejigu, Tariku Nigatu, Eden Dagnachew Zeleke

**Affiliations:** Department of Nursing, College of Health Science and Medicine, P.O. Box 419, Dilla University, Dilla, Ethiopia; Department of Public Health, College of Health Science and Medicine, P.O. Box 419, Dilla University, Dilla, Ethiopia; Department of Nursing, College of Health Science and Medicine, P.O. Box 419, Dilla University, Dilla, Ethiopia; Department of Nursing, College of Health Science and Medicine, P.O. Box 419, Dilla University, Dilla, Ethiopia; Department of Midwifery, College of Health Science and Medicine, P.O. Box 419, Dilla University, Dilla, Ethiopia; Department of Nursing, College of Health Science and Medicine, P.O. Box 419, Dilla University, Dilla, Ethiopia; Digital Health Activity, John Snow, Inc., Boston, MA, USA; Ohio State Global One Health Initiative, Addis Ababa, Ethiopia

**Keywords:** active participant-centred reporting, adverse event reporting, immunization, pharmacovigilance, surveillance, vaccine

## Abstract

**Background:**

Participant-centred active adverse event following immunization (AEFI) surveillance can offer real-time vaccine safety data and help in signal detection. This study aimed to evaluate the effectiveness of participant-centred active adverse events (AEs) surveillance following measles immunization in Gedeo Zone health facilities in Ethiopia.

**Methods:**

An open-label, multicentred, three-arm randomized uncontrolled trial was conducted from 1 June to 21 October 2023. After assessing enrolment eligibility, the study participants were randomized into three groups (short message service [SMS], telephone interview, and diary card). They were expected to report AEs in children 1 week after receiving immunization. Binary and multivariable logistic regression and χ^2^ tests were used to analyse the data.

**Results:**

Among the 396 participants randomized into the three groups, 80.8% (320 participants) reported back about their children's AE status. Participants in the telephone interview group exhibited a substantially superior response rate (93.2% of 132 participants; p<0.00001) compared with the SMS (71.2%) and diary card (78%) groups. The likelihood of reporting the status of AEs experienced by children was lower by 77% (adjusted odds ratio 0.23 [95% confidence interval 0.1 to 0.52], p-value <0.00001) in the diary card group compared with the telephone interview group.

**Conclusions:**

In this study, a telephone interview was found to be the best method for AEFI reporting. Participant-centred active AE surveillance could potentially permit more rapid identification of emerging safety signals.

**Trial registration:**  https://clinicaltrials.gov/ct2/show/NCT05803538.

## Introduction

The measles vaccine is a safe and effective live attenuated vaccine (LAV) that is given subcutaneously on the left upper arm. Reports indicate that there has been an increase in adverse events following immunization (AEFIs), including that of measles vaccine during mass campaigns, due to an increased number of doses administered over a short time and vaccination of an older, wider age group.^1^ An AEFI is ‘any untoward medical occurrence which follows immunization and does not necessarily have a causal relationship with the usage of the vaccines’.^2^ Vaccine pharmacovigilance is the science of the detection, assessment, understanding, taking action and communicating AEFIs or immunization-related issues.^3,4^ AEFI surveillance (active and passive) can deliver reliable data and communicate updated information on the benefit–risk profile of vaccine safety, potentially preserving public trust in vaccinations.^5^

Vast novel methods for AEFI surveillance have been practiced by high-income and developing countries, including vaccine data linking systems,^6,7^ cohort event monitoring,^8^ and sentinel site AEFI surveillance.^5,9,10^ These novel methods used electronic health data,^6,7^ self-reported AEs to trained supervisors and medical officers^8^ and AEs reported from public and private tertiary care hospitals and clinics^5,9,10^ as a source of AEFI data. However, with their limitations, such high resource requirements and expertise, attention has shifted to methods that involve vaccine recipients.^11^ However, active surveillance has the capability of identifying more AEFIs, especially those presenting to sentinel sites, like hospitals, and can compare the rate of AEFIs based on vaccination status and temporality, unlike passive surveillance systems, which encounter drawbacks such as unverified diagnoses, scarce reporting of severe adverse events data, limited elaboration of a temporal link between AEFIs and vaccination and implausible reporting of delayed adverse events.^12^

Countries with functional vaccine safety surveillance systems are expected to record at least 10 reported AEFIs per 100 000 surviving infants annually.^13^ However, for Ethiopia, this corresponds to only 0.0868 AEFIs per 100 000 surviving infants in 2019, 2 AEFIs per 100 000 surviving infants in 2018 and 13 AEFIs per 100 000 surviving infants in 2017.^14^ A total of 53 AEFI reports were made in 2018 at the national level. These AEFIs were detected during measles supplementary immunization activities and the human papillomavirus vaccine (HPV) campaigns. This finding suggests that reporting of AEFI cases during routine vaccination is still challenging in Ethiopia.^15^ Also, according to a recent system and facility readiness assessment for conducting active surveillance of AEFIs in Addis Ababa, Ethiopia, out of three hospitals they assessed, only one of them scored >50% for readiness to implement active AEFI surveillance.^16^

Participant-centred active AEFI surveillance involves proactive collection of vaccine safety data from users during vaccination campaigns for timely detection of AEFIs and places where no vaccine safety monitoring exists.^17^ It has public trust because of less bias from health professionals, which follows their positive attitude towards vaccines and is an easy and transparent data collection process that enables appropriate and timely public health response. Despite its known advantage in signal detection, it remains underutilized.^11^

Even though other methods of active vaccine safety surveillance have been practiced globally, which can offer rapid real-time data collection and dissemination of risk–benefit profiles with ease of implementation, to date, no study has been conducted on the feasibility of participant-centred active surveillance methods in Ethiopia. This study compares short message service (SMS), telephone interviews and diary cards in active adverse event surveillance following measles immunization.

## Methods

### Study procedure

An open-label, multicentred, three-arm randomized uncontrolled trial was conducted from 1 June to 21 October, 2023 to evaluate the effectiveness of SMS, telephone interviews and diary cards for reporting participant-centred adverse events following measles immunization in Gedeo Zone health facilities in Ethiopia. This study was conducted in three healthcare facilities (two health centres and one general hospital). On average, each of the two health centres manages roughly 30 outpatient visits per day, while the general hospital handles approximately 116 outpatient visits per day.^18^

Gedeo Zone is part of South Ethiopia Regional State in southern Ethiopia. It was formed from the southern part of the Southern Nations, Nationalities and Peoples’ Region (SNNPR) on 19 August 2023 after a successful referendum. The region's political and administrative centre is Wolaita Sodo. Notably, six regional bureaus have been established in Wolaita Sodo, Dilla, Arba Minch, Sawla, Karati and Jink.

Study participants (parents, caregivers or guardians) were recruited when they came to health facilities in the zone to get their children immunized for measles. Eligibility criteria for enrolment into the study included mobile phone ownership or access to a mobile phone within the household; ability to read and write or someone in the household who can read and write; being the parent, caregiver or guardian of the indexed child that received the measles vaccine; age >18 years; and willingness to participate in the study and give informed consent (Appendix 2).

### Randomization

After enrolment in the study, participants were randomized either to SMS, telephone interview or diary card methods using a six-block randomization design. The randomization sequence was concealed using sealed envelopes.

#### SMS

Those participants who were randomized to the SMS group were sent text messages through SMS on day 7 following immunization to request whether their children had experienced any adverse event. Those who replied ‘yes’ via SMS were requested to send their answers to a list of questions we provided them during enrolment.

#### Telephone interview

This group received telephone calls on day 7 following immunization and participants were interviewed about whether their children experienced any adverse event. If the participant responded ‘yes’, additional information was sought about the AEFI during the call.

#### Diary card

People in this group received diary cards from the health facility upon vaccination to keep a record of adverse events they observed in their immunized children. Seven days after vaccine administration, parents, caregivers or guardians brought the diary cards back to the health facility, where study nurses collected the diary cards from the participants.

### Statistical analysis

The data were entered into EpiData version 3.3.0 and analysis was done using the R-Studio software version 4.3.1 (Posit, Boston, MA, USA). Intention to treat was used to analyse the data from the participants randomized into the three groups. Descriptive statistics were used to summarize the data. The likelihood of reporting an AE was compared across the participants in the three arms. Binary and multivariable logistic regression and χ^2^ tests were used to analyse the data. A cut-off point of 5% significance and 95% confidence level were used to determine statistical significance.

The effects of the interventions were adjusted for baseline measurements such as the study participant's gender, age, educational status, marital status, employment status, and distance from the health facility. Additional variables included the child's history of adverse events within 7 days after immunization, severity of AEs and presence or absence of AEs within 7 days. We assigned only one AE per child (e.g. child with fever and fatigue was assigned fever, child with fever and swelling at the injection site was assigned swelling). In the education section, we prepared a separate category for those participants who are able to read and write but do not have a formal education, as some people in Ethiopia learn how to read and write in church.

## Results

**Figure 1.**
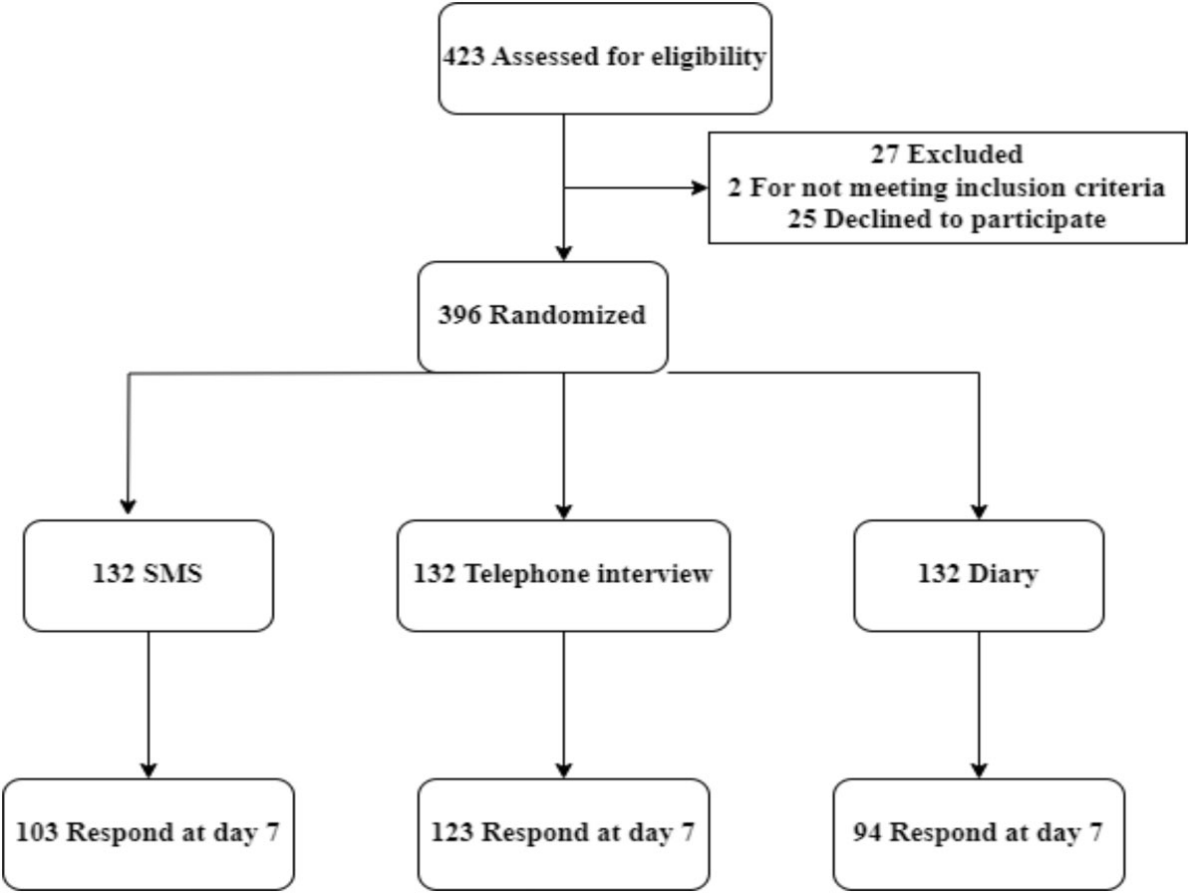
Consolidated Standards of Reporting Trials diagram for a randomized controlled trial of participant-centred active surveillance for AEs following measles immunization.

Between 1 June and 21 October 2023, 423 individuals were approached in three healthcare facilities (one hospital and two health centres) in Gedeo Zone, Ethiopia. A total of 396 participants were randomized to the SMS, telephone, and diary card arms (Figure 1).

Participants across study arms exhibited comparable characteristics. Caregivers were predominantly women (88.4% [n=350]), with a majority identifying as housewives (55.3% [n=219]). The mean distance of the participants’ residence to the health facilities was 2.4 km (standard deviation [SD] 1.9) (Table 1).

**Table 1.** Demographic details and response of individuals (N=396) who participated in the participant-centred active AEs surveillance following measles immunization

Participant demographics and response summary					
		Intervention allocation, n			
		SMS	Telephone	Diary	Total, n (%)
Sex of the child	Male	60	69	73	202 (51)
	Female	72	63	59	194 (49)
Sex of the caregiver	Male	17	17	12	46 (11.6)
	Female	115	115	120	350 (88.4)
Marital status	Married	129	130	129	388 (98)
	Single	2	0	1	3 (0.8)
	Divorced	1	1	2	4 (1)
	Separated	0	1	0	1 (0.3)
Level of education	Unable to read and write	4	16	14	34 (8.6)
	Able to read and write	19	18	12	49 (12.4)
	Primary cycle (1–4)	2	2	9	13 (3.3)
	Secondary cycle (5–8)	8	21	27	56 (14.1)
	High school (9–10)	24	35	42	101 (25.5)
	Preparatory (11–12)	25	17	6	48 (12.1)
	Tertiary education	50	23	22	95 (24)
Employment	Unemployed	3	7	3	13 (3.3)
	Self-employed	37	28	17	82 (20.7)
	Government employee	41	19	16	76 (19.2)
	Non-government organization	2	0	0	2 (0.5)
	Housewife	47	77	95	219 (55.3)
	Student	2	1	1	4 (1)

### AEFI status reporting

Of the 396 participants randomized into the three arms, 320 (80.8%) reported the AEFI status of their children 1 week following immunization. The reporting of AEFI status was higher in the telephone interview group (93.2% of 132 participants) compared with the SMS (71.2% of 132), and diary card (78% of 132; p<0.00001) groups.

The likelihood of reporting the status of an AE experienced by children was lower by 77% (adjusted odds ratio [AOR] 0.23 [95% confidence interval {CI} 0.1 to 0.52], p<0.00001) in the diary card group compared with the telephone interview group. The likelihood of AE status reporting was 4.8 times higher (95% CI 1.37 to 16.95; p=0.015) among participants who can read and write compared with those who can't (Table 2).

**Table 2.** Response proportion and associated factors following measles immunization

	AEFI response status, n (%)			
Variables	Yes	No	Adjusted odds ratio (95% CI)	p-Value
SMS	94 (71.2)	38 (28.8)	1	
Telephone interview	123 (93.2)	9 (6.8)	1.47 (0.78 to 2.74)	
Diary card	103 (78)	29 (22)	0.23 (0.1 to 0.52)*	<0.00001
Level of education				
Unable to read and write	30	4	1	
Able to read and write	31	18	4.81 (1.37 to 16.95)*	0.015
Primary cycle (1–4)	12	1	0.41 (0.04 to 4.14)	
Secondary cycle (5–8)	45	11	1.66 (0.46 to 5.94)	
High school (9–10)	78	23	2.03 (0.62 to 6.63)	
Preparatory (11–12)	40	8	1.57 (0.40 to 6.16)	.
Tertiary education	84	11	0.85 (0.24 to 3.05)	

*In a regression table indicate the level of the statistical significance of a regression coefficient.

### AEs

A total of 52 AEFIs were reported, with fever being the most widely reported AE (42%). Most (94.2%) AEs were characterized as mild. Among participants experiencing AEs, the mean duration for the onset of the first AE was 2 d (SD 1.2). The mean duration for the resolution of AEs was 3 d (SD 1.8). Notably, a substantial portion of study participants (31 of 41) expressed a preference for administering home remedies in response to AEs (Table 3).

**Table 3.** AEs following measles immunization

Variables		AEs, n (%)
AEs (n=52)	Fever	22 (42.3)
	Fatigue	1 (1.9)
	Rash	4 (7.7)
	Swelling	2 (3.8)
	Redness and pain at the injection	6 (11.5)
	Rigor or convulsion	1 (1.9)
	Other	16 (30.8)
Degree of AEs (n=51)	Mild	49 (94.2)
	Moderate	3 (5.8)
Statutes of AEs (n=51)	Resolved	27 (51.9)
	Recovering	20 (38.5)
	Not resolved	5 (9.6)
Did you take the child to the health facility? (n=51)	Yes	7 (13.5)
	No	45 (86.5)
What kind of treatment did the child get? (n=41)	Treatment from health facility	7 (19)
	Traditional medicine	2 (4.9)
	Home remedies	31 (75.6)

## Discussion

Studies have shown that active surveillance provides a heightened level of monitoring that is more responsive, enabling timely detection of signals and furnishing data for regulatory and public health entities. By conducting direct, near-real-time surveys of consumers and publicly sharing the results, active surveillance systems address transparency issues, and enhance public confidence in the overall immunization program.^11,12^ In various countries, numerous methods have been applied to enable direct data collection from vaccinees, caregivers or guardians, including diary cards^19–21^ SMS,^17,22,23^ telephone interviews,^24–27^ mobile applications, and web-based notification.^28,29^ However, according to recent narrative reviews, most of these studies on participant-centred active surveillance have been conducted in high- and middle-income countries, with limited studies in sub-Saharan Africa.^11^

This study aimed to evaluate the effectiveness of using SMS, telephone interviews and diary cards for participant-centred AEFI reporting in Gedeo Zone, Ethiopia. The majority of the participants (320/396 [80.8%] responded to the study 1 week after vaccination. The comparison of AEFI reporting 1-week post-vaccination revealed a significantly higher percentage of participants reporting in the telephone interview group compared with the SMS and diary card groups. Notably, the scarcity of studies directly comparing telephone interviews with these alternative methods underscores the novelty of our findings. Previous research incorporating telephone interviews, either in isolation or in conjunction with other surveillance methods, has demonstrated their efficacy in enhancing vaccine safety data collection.^24^ This approach facilitated the identification of mild and self-limited AEFIs while being well-received by study subjects.^30^

Moreover, the consistent pattern observed in various studies suggests that telephone interviews often yield higher response rates, indicating a more robust and reliable data collection process.^24,25,27,31^ The direct interaction inherent in telephone interviews allows for immediate clarification of queries, ensuring accurate and comprehensive reporting. Additionally, the qualitative insights derived from such interviews contribute to a more holistic understanding of AEs. However, it is essential to acknowledge the potential limitations of this approach. The practicality of employing telephone interviews may be compromised in developing countries or areas with limited telephone us. This method also demands substantial resources, including manpower and time.^32^ Furthermore, responses may be susceptible to social desirability bias, as participants may exhibit a tendency to provide answers perceived as socially acceptable.

Our study findings reveal a lower probability of AE reporting using SMS compared with telephone interviews. Interestingly, this contradicts the growing global evidence supporting the feasibility of SMS as a robust method for data collection. Previous studies have demonstrated that automated SMS-based reporting can establish sustainable, real-time monitoring of AEs, contributing to the early identification of potential vaccine safety issues.^22,33–36^ While SMS offers a more cost-effective alternative to telephone interviews, demanding fewer resources, and allowing for swift participant responses, it presents unique challenges. The brevity inherent in SMS communication imposes limitations on the amount of information conveyed, potentially resulting in incomplete reporting. Additionally, some participants may encounter technical difficulties or express discomfort with text messaging, thereby influencing response rates. Another critical factor is the familiarity of study participants with text messaging and their level of literacy, which could further impact the effectiveness of SMS-based reporting.

In diverse settings such as Ghana, Italy and Taiwan, the utilization of diary cards has been implemented, providing parents/guardians with a tool to record AEFIs. This approach offers distinct advantages, allowing for the collection of AEFIs over an extended period. Diary cards enable the observation of trends and patterns, empowering participants to furnish detailed information, and contributing to a more nuanced understanding of AEs.^20,21,32^ However, the inherent nature of diary entries introduces potential limitations. Unlike real-time methods such as telephone interviews or SMS, diary entries may lack immediacy, potentially impacting the timeliness of signal detection. The effectiveness of diary cards may also be influenced by the level of literacy among participants, as the interpretation and accurate recording of events rely on written communication skills. Additionally, participants may face challenges in consistently updating their diaries, leading to potential gaps in the data collected over time.

While telephone, interviews may offer robust data with high response proportion, SMS, and diary methods present more cost-effective and participant-friendly alternatives. Combining multiple methods or employing a hybrid approach might enhance the strengths of each method while mitigating their respective limitations in comprehensive AE surveillance. Despite these considerations, our study contributes valuable insights into the comparative effectiveness of different surveillance methods for AEFI reporting. Future research should explore the feasibility and limitations of these methods in diverse settings to inform optimal strategies for vaccine safety monitoring.

### Limitations

Owing to the limited participant enrolment, the study faced limitations in statistical power, hindering the ability to draw meaningful conclusions from the findings. Moreover, the absence of blinding in the study design could potentially influence participant selection. The inclusion of a small participant pool was necessitated by limited funding, potentially affecting the generalizability of outcomes, the detection of significant relationships or differences and the exploration of subgroup variations within the data. Despite efforts to address missing data and maintain consistent follow-up across intervention groups, the possibility of selection bias remains a concern.

## Conclusions

Participants in this study were significantly more likely to respond to a telephone interview–based safety survey rather than SMS and diary cards. Participant-centred active AE surveillance could potentially permit more rapid identification of emerging safety signals.

## Data Availability

Data are available upon request from the corresponding author.

## Appendix 1. Questionnaire

**Table:**

No.	Variable	Coding categories
1	Sex	1. Male 2. Female
2	Age (in years)	–––––
3	What is your religious affiliation?	1. Orthodox 2. Muslim 3. Protestant 4. Catholic 5. Others, please specify –––––
4	Marital status	1. Married 2. Single 3. Divorced 4. Widowed 5. Separated
5	Highest educational qualification	1. No read and write 2. Read and write 3. 1st cycle (1–4) 4. 2nd cycle (5–8) 5. High school (9–10) 6. Preparatory (11–12) 7. Tertiary education
6	How far are you from this facility? (in km)	–––––
7	Residence	1. Urban 2. Rural
8	Employment status	1. Unemployed 2. Self-employee 3. Government employee 4. Non-governmental organization 5. Housewife 6. Student 7. Retired 8. Other, please specify –––––
9	Address	
10	Phone number	
Vaccinee sociodemographic characteristics		
1	Sex	1. Male 2. Female
2	Age (in month/year)	–––––
Vaccination information		
3	What type of vaccine the child received?	1. Measles
4	Route of administration	1. Subcutaneous
5	Intervention allocation	1. SMS 2. Telephone interview 3. Diary card
Questions for next follow-up		
1	Has your child experienced adverse events after his/her immunization?	1. Yes 2. No
2	If yes, can you please choose the adverse events that your child experienced after his/her immunization until this day?	1. Fever 2. Headache 3. Fatigue 4. Rash 5. Swelling 6. Redness, or pain at the injection site 7. Rigors, or convulsions 8. Others, specify ––––
3	When did the listed AEFIs start? (for every AEFI)	
4	How do you describe the adverse events?	
5	What is the status of the AEFI?	1. Recovered/resolved 2. Recovering 3. Not resolved/ still occurring
6	How long did it stay with your child?	
7	Have you visited a health facility for your child?	1. Yes 2. No
8	If yes, where did you go?	1. Health centre 2. Hospital 3. Emergency department 4. Traditional healer
9	What type of treatment have you gotten for the problem?	1. Treatment prescribed from hospital 2. Traditional medicine 3. Home remedies
		**Thank you!!!**

## Appendix 2. Information sheet and informed consent

My name is –––––––– and I am Nurse/Midwife. I am working as study nurse for the project titled ‘Participant Centered Active Surveillance for Adverse Event Following Measles Immunization in Gedeo Zone, Ethiopia, 2023. A multicentre open-label randomized control trial’. The project aims to study the feasibility of SMS, telephone interview and diary for reporting of AEFI's on active surveillance. You have been invited to take part in this activity because you are parent/Guardian/care giver of the child that has received vaccine.

**1.** **Why is this evaluation being done?**

The national immunization program delivers effective and safe vaccine. However, adverse events might occur after immunization due to many reasons. The Ethiopian Pharmacovigilance centre which is under Ethiopian Food and Drug Authority (AEFI) has the mandate to control safety of medicine including vaccine and have the system to spontaneously report every AEFI's encountered by vaccinee. However, Ethiopia doesn't reach the recommended rate of AEFI's per 100 000 surviving infants to be reported to WHO through WHO UNICEF/Joint reporting form (JFR) through passive reporting. We aimed to study possible alternative participant centred active surveillance method which can complement the routine surveillance system and help in signal detection.

**2.** **What type of information will be collected?**

If you decide to participate, we expect that you will be in this activity for one week. During this time, you will be required to provide information on scheduled period through SMS/phone call/Diary card about AEFI encountered by your child after vaccination.

**3.** **What are the risks and benefits for participants?**

There are no direct benefits to you from your participation in this research. However, possible benefits to others may include providing the study result for policy makers and different stake holders to improve the vaccine safety surveillance system.

Participation is completely voluntary. You can decide to participate or not to participate. **Your alternative to participating in this activity is not to participate.** If you decide to participate, you will be required to provide your consent be signing an informed consent form.

**4.** **How will my privacy be protected?**

All information you provide will be kept confidential. Only authorized study nurse and investigators will have the access to the information you have given us.

### Informed consent form

**herewith declare that**
✓ The objectives of this study are explained to me and are clear.
✓ The contents of the consent are verified to me to participate in the study.
✓ I acknowledge that if the research is published it will not include my name.
✓ If research is published the data will be grouped so that I cannot be personally identified.
✓ I may decide at any stage, without prejudice, withdraw my consent.
✓ I had enough time and a chance to ask questions.
✓ I am willing to take part in this research project.
✓ I understand that I can say no to research and that my care at the clinic will not be affected.

I understand that participation in this study is completely voluntary and that I may withdraw at any time without supplying reasons. I agree to participate in this study to be interviewed, provided my privacy is guaranteed. When signing this consent form to participate in the study, I promise to answer honestly to all reasonable questions and not provide any false information or in any other way purposely mislead the researcher.

PARTICIPANT:

Printed Name and Surname: _______________________________________

Signature/Mark or Thumb print: ____________________________________

Date: _______________________

WITNESS (If applicable):

Printed Name and Surname: _______________________________________

Signature: _________________________________________________

Date: _______________________

I, ______________________ (insert name of research staff), herewith confirm that the above participant has been fully informed about the nature, conduct and risks of the above study.

STAFF TAKING INFORMED CONSENT

Printed Name and Surname:

Signature: _____________________________________________________

Date: _____________________________

Script to be used after registration to inform participants about scheduled follow up visits

- Thank you for participating in this study.
- We will contact you on day seven to ask how your child have been feeling since taking immunization through SMS/telephone interview and you are expected to return the diary card for the examining nurse on day seven (for participants on diary card group).
- If you have any concerns after your immunization, we encourage you to come back to this site to speak with a nurse or doctor about it.
